# Abattoir-Based Measures to Assess Swine Welfare: Analysis of the Methods Adopted in European Slaughterhouses

**DOI:** 10.3390/ani11010226

**Published:** 2021-01-18

**Authors:** Silvio De Luca, Emanuela Zanardi, Giovanni Loris Alborali, Adriana Ianieri, Sergio Ghidini

**Affiliations:** 1 Department of Food and Drug, University of Parma, Via del Taglio 10, 43126 Parma, Italy; emanuela.zanardi@unipr.it (E.Z.); adriana.ianieri@unipr.it (A.I.); sergio.ghidini@unipr.it (S.G.); 2 Istituto Zooprofilattico Sperimentale della Lombardia e dell’Emilia Romagna-Headquarters, Via A. Bianchi, 9, 25124 Brescia, Italy; giovanni.alborali@izsler.it

**Keywords:** welfare indicators, abattoir, animal-based measures, scoring schemes

## Abstract

**Simple Summary:**

The welfare of pigs is a major concern among some consumers and pig producers. This concern has driven welfare specialists into the validation of methods and protocols that can be used to evaluate the welfare status of pigs on farms. These protocols require the use of animal-, management and resource-based measures, although data collected from the animals are generally considered more useful. However, due to some limitations, these schemes are not always applicable; therefore, the use of simplified schemes involving the collection of data from pigs at the slaughterhouse has been advocated. Methods and scoring schemes recently described and used in studies performed in European slaughterhouses to determine pig welfare and health are reviewed in the present manuscript. A focus on the scoring schemes for lesions of the body and viscera during post-mortem activities at the abattoir is provided. These methods can be used to benchmark a welfare scheme suitable for all European competent authorities and professionals working in the context of pig welfare.

**Abstract:**

The assessment of swine welfare requires feasible, reliable, and reasonable indicators. On-farm evaluation of pig welfare can provide valuable information to veterinarians and farmers. However, such protocols can result expensive and time-consuming. With this regard, an interest in the appraisal of swine welfare at abattoir has grown over the recent years. In particular, the use of certain lesions collected directly from slaughtered animals to determine the welfare status of pigs has been evaluated by several authors. In the present review, the different methods developed to score lesions collected directly from the body and the viscera of animals slaughtered in European abattoirs (“abattoir-based measures”) are presented. The text specifically focuses on the methods currently available in the literature for the scoring of body, pluck and gastric lesions during post-mortem activities. Moreover, the strengths and weaknesses of abattoir-based measures schemes are discussed. To conclude, the future perspectives of the assessment of pig welfare at the slaughterhouse are described, appealing for a benchmarking system that can be systematically used by veterinarians and other professional figures involved in the process.

## 1. Introduction

Over the past years, consumers and stakeholders in Europe increasingly recognize the concept of animal welfare, with a special interest towards the pig industry, as a benefit to the meat market and a general attribution of food quality [1,2]. The increased awareness of the importance of swine welfare in consumers has motivated the development of market-driven strategies towards elevated animal welfare standards on farms [3,4]. In Europe, the welfare of pigs is included in a large body of legislation, which sets specific rules and minimum standards for the protection of pigs on farms, during transport and at slaughter [5,6,7]. Regulation (EC) 625/2017 contains requirements concerning the official controls in the matter of animal welfare. In particular, article 21 appeals to “*the use of specific animal welfare indicators […] and the design of such indicators on the basis of scientific and technical evidence*” [8]. There has been a growing general interest in the identification of such indicators and their inclusion in a monitoring system in order to assess swine welfare on the farm and at the slaughterhouse [9,10,11]. An example is the application of the Welfare Quality^®^ protocols [12,13] that allow a standardized assessment of animal welfare on pig farms through education and specific training of qualified welfare auditors [14]. These protocols include a) resource-based measures, which are collected from the environment surrounding the animals, e.g., the number of drinkers; b) management-based measures, which take into account the way how the animals are managed, e.g., the pen density; c) animal-based measures (ABMs), which are collected directly from the animal, e.g., the body condition score or the numbers of wounds on the body [15]. Nevertheless, Welfare Quality^®^ protocols focus more on ABMs since these measurements can better describe how an animal reacts to the surrounding environment [14,16]. Although such methods are considered reliable and repeatable [17], their use for the assessment of swine welfare may result expensive and time-consuming [18]. Thus, a general interest in the value of welfare outcomes collected directly from swine carcasses (“abattoir-based measures”) as indicators of on-farm health and welfare has lately arisen [10,19,20,21].

Detection and evaluation of lesions correlated to animal welfare issues may be included in the standard meat inspection (MI) activities, thus expanding its role as a health and welfare monitoring tool, as also appealed by article 21 of the Regulation EC 625/2017 [8,22]. In particular, certain lesions such as bites, scars or necrosis located on the tail and the skin together with the presence of bursitis are considered as “iceberg indicators” of welfare problems on pig farms [19,23,24].

In fact, these types of lesions, developed during the rearing cycle, can represent a warning signal for different welfare issues, such as resting or thermal discomfort, among others [24]. Moreover, such lesions can be practically recorded during post-mortem MI activities and eventually, they may be embodied in a recording system for pig health and welfare monitoring purposes together with other organs score (e.g., pluck lesions). Of note, the detectability of certain lesions, such as tail or skin lesions, increases after scalding and dehairing of carcasses [25,26]. Therefore, the precise moment in which the record is performed is crucial.

Other lesions rather than body (e.g., tail necrosis or wounds) and pluck (e.g., pleurisy or pericarditis) lesions may be indicative of welfare issues. Among these, gastric lesions have been related to several stress-factors such as pen density, floor type, transport and harsh environmental conditions [27]. Therefore, these lesions may be included in the context of the abattoir-based measures because they can be visually evaluated by the assessors throughout slaughtering procedures.

Taking into account the variety of approaches described to date in the literature to assess swine welfare at the abattoir, even though the parameters evaluated have common features between them, the aim of this review is to address the methods commonly used in European pig slaughterhouses intended to collect abattoir-based measures. In particular, the text focuses on the scoring methods most frequently employed during the post-mortem inspection activities, with a special emphasis on the step of the slaughtering process that they are applied. In the following sections, the scoring and evaluation systems for the body, pluck and gastric lesions, respectively, are described. Lastly, the strengths, weaknesses of the scoring methods as well as the future perspectives of the schemes based on abattoir-based measures are also discussed.

## 2. Body Lesions

Reliable scientific evidence is available for some body lesions such as tail and skin lesions and, partially, bursitis. On the other hand, information is lacking on other conditions that can be evaluated at the slaughterhouse, such as dermatitis [20] and decubital ulcers [28]. Therefore, such lesions are not further discussed.

### 2.1. Tail Lesions

Tail lesions are the consequence of tail biting and abnormal behavior displayed by domestic pigs through biting or chewing the pen mates’ tails [29]. Such a problem is widely diffused in the pig industry [30,31]. Different environmental and/or management conditions are involved in the triggering of this aberrant behavior, such as the incorrect distribution of the pigs within the pens during the weaning phase, presence of high levels of ammonia in the air or irregular feeding habits [31]. In particular, the lack of human interventions aimed to isolate aggressive piglets from the pen or the lack of chewable material are considered major risk factors linked to tail biting behavior [32,33].

It has been shown that improvement in the farm management (e.g., reducing the percentage of pens with fouled bedding or with wet/damp lying areas), and the enrichment of the environments (e.g., providing raw material such as corn silage since the first weaning phase) may reduce the episodes of tail biting [34,35,36]. Tail lesions may range from mild punctures to severe wounds, necrosis and partial or total amputation of the tail [37]. These lesions can be the entry route for pathogens and subsequent dissemination through the bloodstream in different organs [38].

Different authors have found a direct correlation between the presence of severe tail lesions at the slaughterhouse and several pathological conditions, such as pleurisy or pneumonia [10,18,19,20,21,39], increased number of whole carcass condemnation, loin bruising and bursitis [19], as well as decreased carcass weight [9].

A few systems, feasible for commercial abattoirs for the scoring of tail lesions, have been proposed and validated. One of the firsts was proposed by Kritas and Morrison [40] and then partially adapted for further studies by other authors [10,39]. This scoring system is based on a 5-point scale, going from 0 (“no lesions”) to 4 (“partial or total loss of tail”) (Table 1), and it has been used in a number of studies [9,10]. Following this scheme, van Staaveren et al. [39] adopted and modified this scoring system, splitting the last score value (score = 4) into two further scores (4 and 5), where the score equal to 4 is defined as “partial loss of the tail”, and the score equal to 5 is defined as “complete loss of the tail” (Table 1).

A second scheme, using a 6-point scale system, was proposed by Keeling et al. [41] (Table 1). In a study performed in two Swedish slaughterhouses, the authors found differences between observers when the scoring results were compared, despite such differences were mainly attributed to disagreements in the assessment of the score 2 (“small sore or wounds”).

A third scoring system, based on a 5-point scale going from 0 (“no lesions”) to CL (“complete loss of the tail”), was developed by vom Brocke et al. [30] (Table 1) to determine tail lesion prevalence and the association between MI findings and tail lesions in a commercial abattoir located in Germany. No data of comparison between this system and the methods proposed by Kritas and Morrison [40] and Keeling et al. [41] are currently available.

### 2.2. Skin Lesions

As per tail lesions, skin lesions are a valuable source of information concerning the welfare of pigs on farms [17]. The latter lesions are generally linked to aggressive behavior between pen mates and inefficient management at the farm level [43]. Among others, the regrouping of pigs during the rearing phase [43], the feeding regime [44], and the pen density [45] are considered on-farm risk factors favoring skin lesions, although other factors are involved in the development of skin lesions, such are transport and lairage at the slaughterhouse [46,47,48].

The Welfare Quality^®^ protocols for pigs include the assessment of skin lesions at the slaughterhouse [12] (Table 1). In this case, the evaluation of skin lesions needs to be performed on one side of the selected carcasses and addressed to 5 parts: 1—ears, 2—front (from the head to the back of the shoulder), 3—middle (form the back of the shoulder to the hindquarters), 4—hindquarters and 5—legs (from the accessory digit upwards). Each of these parts is then scored using a 3-point scale, as described in Table 1. This scoring system provides good information about skin lesions; however, it may result time-consuming and therefore not feasible for commercial slaughterhouses [42]. In an attempt to overcome this issue, two adapted versions of the system, feasible for high-speed lines, have been proposed.

First, Bottacini et al. [23] suggested a method based on a 3-point scale going from 0 (“no lesions”) to 2 (“more than 5 lesions”) that contemplates the pig carcasses to be divided into two parts, one caudal, corresponding to the hind legs and the tail, and one cranial, including the rest of the body (Table 1).

Second, the size and severity, instead of the number, of lesions was considered in the system developed by Aaslyng et al. [42], who proposed a 4-point scale, going from 0 (“no lesions or very small lesions”) to 3 (“much deep damage”).

An alternative method, also feasible for the commercial abattoir, was proposed by Carroll et al. [18]. This method was designed to validate the degree to which skin lesions collected at the abattoir can reflect the on-farm situation. The authors used in the first place a 6-point scoring system to evaluate skin lesions at the farm level (experimental units) on live animals. Then, the previous system was adjusted for scoring skin lesions at the slaughterhouse, condensing it into a 4-point scale, with scores 2 and 3 being classified as “mild” and score 4 and 5 as “severe”. In Table 1, the method used during the rearing phase is included. According to this, the front (ears, snout, shoulders, and front legs), the middle (flank and back) and the rear (hindquarters and back legs) parts are separately assessed. Furthermore, a distinction between fresh (red) and healed (not-red) lesions is included.

### 2.3. Bursitis

Bursitis is fluid-filled sac tissue that develops in the subcutaneous connective after excessive pressure on the skin over a skeletal prominence [14]. These lesions typically arise below the hock joint of the hind limbs and less frequently on the forelimbs [49]. Therefore, bursitis is a good indicator of comfort around resting in pigs [20]. The prevalence and the severity of such lesions on-farm are related to the different types of flooring, the growing stage, and the production systems nowadays present in the pig industry [14]. To the best of our knowledge, only one scoring method for bursitis feasible for studies at the slaughterhouse has been described to date in the literature (Table 1). However, some authors have also collected data about bursitis in pigs at the slaughterhouse, applying a simple binary score in terms of the presence/absence of the lesions [19].

## 3. Pluck Lesions

Pluck lesions are lesions of a group of organs that belong to pigs’ red offal [50]. This section focuses on lesions of the lung and the pleura, which are considered of great importance, while lesions concerning the other organs of the pluck, such as the heart (e.g., pericarditis) and liver (e.g., ascaridiosis), are not included due to the lack of specific scoring methods. However, the evaluation of the presence of these lesions has been included in a few studies [51].

### 3.1. Lung Lesions

Lung lesions are common findings in finisher pigs at the slaughterhouse [52]. In fact, respiratory disease severely affects pig production worldwide [53], and it is related to the porcine respiratory disease complex (PRDC), a multifactorial disease caused by a number of infectious agents, environmental conditions, and management practices [54,55]. Catarrhal bronchopneumonia is typically associated with *Mycoplasma hyopneumoniae*, the primary agent of enzootic pneumonia (EP) [56,57].

EP-like lesions normally appear as a purple or gray area of pulmonary consolidation of the cranioventral lobes [58]. The appearance of the lesions depends on the stage of the disease: damaged lobes are swollen in the acute stage of the disease, while in the chronic phase, interlobular scarring tissue is typically present [59]. The extension to which the pulmonary lobes are affected by the inflammation process can be assessed using different scoring methods.

According to Garcia-Morante et al. [59], the scoring methods for EP-like lesions can be based on either a two-dimensional or a three-dimensional approach [59]. In the first case, the estimation of the damage is proportional to its extension on the lung surface, while in the three-dimensional approach, the relative weight of each lobe is taken into account in the final score [59,60]. The three-dimensional approach systems are less feasible for scoring the lung lesions at the slaughterhouse, therefore, not further considered in the present overview.

The method proposed by Madec and Derrien [61] is often used in studies [55,56,62,63] performed at the slaughterhouse (Table 2). This method (“Madec’s grid”) contemplates the division of each lobe into quarters, followed by the scoring of the lobes by means of four points, based on the extension of the lesions, for a maximum total score of 28 [60,61]. This system allows the evaluation of lung lesions even in high-throughput slaughterhouses. However, Madec’s grid is not able to represent the entire damage affecting the lung since each lobe does not embody an identical proportion of the lung (i.e., the surface of the caudal lobe is greater than the surface of the cranial lobes) [59]. For this reason, an adapted version of Madec’s grid was proposed [64]. In particular, this scheme can be integrated with the quantification of each lobe weight, according to Christensen et al. [65]. In particular, through the combination of these two methods, the evaluation of the entire lung surface can be carried out [64].

Another scoring system feasible for studies at slaughterhouses [53,66,67] is the one proposed by Straw et al. [68]. According to this system, the lung lesions score is equal to the sum of the percentage of the damage of each lobe multiplied by the relative lobe size for a maximum score of 100% (“rule of ten”) (Table 2). This system was further validated by Steinmann et al. [60] in a study performed to evaluate a simplified scoring system based on the “rule of ten” and, therefore, to increase the accuracy of meat inspection data collected in large German slaughterhouses. In this study, the surface extension of each lobe (cm^2^) from five healthy lungs, printed on a cross-section paper with the smallest areal sensitivity of one mm^2^, was calculated. The arithmetic mean and median of the relative lobe surfaces, expressed in percentage of the lung surface, agreed with the subdivision of the lobes and relative size proposed by Straw et al. [68].

An additional scoring method was developed by Goodwin et al. [69]. This is a 55-point lung lesion scheme, which assigns a higher score to the cranial and medial lobes compared to the caudal lobes (Table 2). This scheme is particularly indicated for the scoring of EP-like lesions, and it has been embodied in the British Pigs Health Scheme (BPEX) and Wholesome Pigs Scotland (WPS) scheme [70].

### 3.2. Pleural Lesions

Likewise, EP-like lesions, chronic pleurisy (CP), primarily caused by *Actinobacillus pleuropneumoniae*, are a type of lesion frequently reported in pigs at abattoir [53,55,71].

Some of the risk factors associated with chronic pleurisy are density, herd size, pig health status and mixing of pigs in the finisher stage [71]. Pleurisy lesions typically appear as fibrous or fibrinous pleural adhesion located in a cranioventral position, often associated with severe EP-like lesions, or a dorsoventral position, indicating a possible healing process from a previous pleuropneumonia [55].

While different systems allow the scoring of EP-like, the assessment of pleural lesions is usually performed according to the “slaughterhouse pleuritis evaluation system” (SPES) proposed by Dottori et al. [72]. SPES grid consists of five different scores, from zero (0 = no lesions) to four (4 = severely extended bilateral lesions) based on extension and location of pleural lesions (Table 2). This method allows the scoring of the visceral pleura, and it gives a higher score to the diaphragmatic lung lobes, which are most likely affected during the course of porcine pleuropneumonia [55]. Moreover, it can provide another output, the “*Actinobacillus pleuropneumoniae* index” or APPI [55,72], which is the frequency of pleuritic lesions with a score ≥ of 2 (Table 2).

Di Provvido et al. [73] developed an alternative scoring method for chronic pleurisy, called “pleurisy evaluation on parietal pleura” (PEPP), designed to evaluate the parietal pleura instead of the visceral pleura (Table 2). Scoring points from both carcasses halves of each pig are separately assigned according to the presence and extension of pleural lesions and then summed for a total score for each pig ranging from 0 to 12 (the maximum score for each half is 6) [73]. This method was validated by the contemporary application of SPES and PEPP scoring systems on two hundred sixteen slaughtered pigs, with both methods strongly correlated with each other (r = 0.913, Pearson’s correlation coefficient). An advantage of PEPP score over SPES score is the reduced presence of confounding factors, such as blood smears or blood clots, on the parietal pleura compared to the visceral pleura; on the other hand, the SPES score allows the contemporary scoring of other organs rather than the pleura, such as the lungs and the liver.

Other methods, such are those based on image analysis, are described for the scoring of the pleurisy lesions, but their complexity makes them not feasible for commercial abattoirs [74]; therefore, such methods are not described here.

## 4. Gastric Lesions

Gastric lesions, in particular oesaphago-gastric ulcers (OGU), are important pathological conditions widely diffused within pigs. Gastric lesions occur prevalently in the pars oesophagea, a non-glandular region of the gastric mucosa not protected by mucus [75]. Under certain circumstances, the pars oesophagea may encounter the low pH content of the distal part of the stomach, rich in pepsin and bile, thus resulting in chronic damage and the possible development of hyperkeratosis, followed by erosion and then ulceration [76]. Several causes have been associated with OGUs in pigs [77]. Among these, feeding practices [78,79], management procedures [80,81] and infection with *Helicobacter suis* [76] play an important role in the development of OGUs. Furthermore, the presence of gastric lesions has been identified as potential indicators of chronic intermittent stress [81]. While the scoring of gastric lesions has been widely used to determine the effect of the inclusion of different elements in the diet (e.g., straw) on the prevalence of OGU, the body of recent literature regarding the scoring of gastric lesions in pigs at the abattoir to evaluate animal welfare is rather scarce. Few methods have been described so far to assess gastric lesions. Among these, two scoring systems are feasible for scoring gastric lesions at the slaughterhouse. The first scheme, proposed by Robertson et al. [82], is based on a 4-point scale, going from 0 (“no lesions”) to 3 (“severe ulcer”) (Table 2). Detection of scars, indicating a previous presence of erosion or ulceration, may be included in the scoring scheme as a binary value (presence/absence) [77].

The second scheme, adopted by the Australian Pig Health Monitoring Service (PHMS), was developed and described by Kopinski and McKensie [83]. This system grades the appearance of the gastric mucosa and, similarly to the previous one, is based on a 4-point scale, going from 0 (‘Shiny white squamous epithelium’) to 3 (‘Developed ulcers, hemorrhage and stenosis present’) (Table 2).

The other two systems to score gastric lesions have been described so far in the literature, although few data are in support of their use at the abattoir. The percentage of the damaged surface is the factor included in the scheme of Hessing in 1992 [84], based on a 6-point scale going from 0 (“normal mucosa”) to 5 (“hyperkeratosis with many erosions or ulceration”). A 6-point scale scheme going from 0 (“no lesions”) to 3 (“gastric ulceration in the pars oesophagea and/or stricture at the cardia”) was also proposed by Grosse Liesner [85] (Table 2).

## 5. Strengths and Weaknesses of Abattoir-Based Measures

The concept of integrating welfare outcomes collected from pig carcasses into routine MI activities is relatively recent [22]. MI was, in fact, primarily recognized as a tool to identify meat unfit for human consumption, while in recent years, experts in the field of animal welfare have explored its potential as a surveillance tool for animal health and welfare [22]. The fact that in Europe, pigs slaughtered for meat are legally required to undergo a MI process puts slaughterhouses at a strategic point along the food chain [11]. This strategic position allows the assessment of an elevated number of pigs from different farms and different producers in a short time and with a relatively lower cost compared to on-farm assessments [18]. Therefore, approaches based on abattoir measures for the determination of pig welfare may reduce the need for on-farm visiting [24], in the view of the fact that on-farm assessments can be furthermore limited by other issues, such as high stocking densities, pigs dirtiness and poor visibility [86]. At slaughterhouses, on the other hand, pigs are subjected to washing and dressing steps prior to post-mortem inspection that are able to increase the visibility of certain lesions [25]. It should be noted that the current legislation states that lighting at abattoirs shall be adequate and sufficient to perform MI activities [87], thus permitting an easier assessment of some lesions such as tail and skin lesions when compared to on-farm evaluations of the same lesions [86]. As already mentioned, the inspection of offal and carcasses are mandatory for pigs destined for human consumption, meaning that a monitoring system based on outcome indicators could be integrated into an already pre-existing MI system [22]. Data collection at this level may benefit the fact that at meat inspection points measures can be collected simultaneously from carcasses and corresponding offal [51]. This is important in the view of the fact that some conditions, such as tail lesions and respiratory diseases, can share the same risk factors like frequent mixing or moving, stocking density, and ventilation [9].

Different European countries have already adopted abattoir inspections-based systems at the national level as a tool for monitoring pig health and welfare. For example, in Denmark, a scheme called the “Danish swine slaughter inspection data system” has been established since 1964 [11]. Similar health schemes have been introduced and implemented in the Netherland, Scandinavia, and Northern Ireland [51,88,89]. Another example of an integrated monitoring scheme based on abattoir measures is provided by the BPEX and WPS, applied in Great Britain and Scotland respectively [51]; these two schemes allow collecting information concerning respiratory diseases (e.g., pleurisy or EP-like lesions), tail damage (presence/absence), peritonitis, pericarditis and conditions affecting the liver [51]. In Italy, a system called “CLASSYFARM” has been recently introduced; this innovative system permits to collect ABMs on the farm and the slaughterhouse, thus allowing the monitoring of health and welfare indicators along with the pig production chain as a whole [90]. All these systems are computer-based and can gather the information that is then communicated to pigs producers and veterinarian advisers, which can use this information to address problems on farms and to evaluate specific interventions such as vaccination programs or improvements in the managing systems [51,91]. Data from abattoirs have also been included in specific management tools developed by farm advisory services to help pig producers reducing some welfare issues, in particular, tail biting. Examples of these services are the German “Schwanzbeiϐ-Intervention program” (SchwIP) and the Irish Teagasc eProfit Monitor [21,30]. Recent studies have shown that these husbandry tools could effectively help farmers that were active participants of the advisory services had a lower prevalence of tail lesions when compared to farms that were not enlisted [21,30]. In this view, this could be of particular interest for pig producers, considering the economic impact that certain welfare-related conditions may have. For instance, it has been estimated that tail lesions can cost up to €1.10 per slaughtered pigs, due to partial or total condemnation of carcasses [19], while pigs with EP-like lesions and pleuritis may account for a post-trimming reduced carcass weight up to 1.26 kg and 1.24 kg, respectively [70].

Although animal welfare assessments based on abattoir outcomes have some advantages, on the other side, such systems may harbor some limitations [22]. These limitations are caused by different factors, such as the applications of different scoring systems that make difficult the comparison between multiple studies, the differences between countries in the application of animal welfare policies, and the applicability of some methods in high-speed slaughter lines [10,19,26,92]. As already mentioned, the use of MI data as indicators of animal welfare has been recently introduced. Hence there are few studies at the moment that compare the scoring methods between them. For instance, Garcia-Morante et al. [59] compared some of the scoring methods described in the literature to evaluate lung lesions. In this study, the authors applied eight lung lesions scoring methods, among which are present those described in the present review [61,65,68,69], to evaluate the extension of pulmonary damage in pigs experimentally inoculated with *M. hyopneumoniae*. A regression analysis applied to compare the different methods revealed a good correlation (r > 0.9) between the diverse scoring systems in terms of evaluation of lung damage extension. However, it should be noted that this study was performed in experimental conditions, and scoring was performed by a trained pathologist veterinarian. Therefore, further investigations should be performed at the abattoir, taking into account factors such as the speed of the line and the desired degree of detail. Apart from the differences in the scoring methods used to evaluate pulmonary lesions, other factors are important in the interpretation of the abattoir-based health and welfare assessment. Among these, the age of the pigs at slaughter and seasonality are other important elements in the definition of the health status of the pigs. For instance, pigs slaughtered at 160–170 kg and 9 months of age (“heavy pigs”) tend to have fewer respiratory lesions compared to pigs slaughtered at 100 kg (“baconers”), probably due to the effect of a healing process [62], while it seems that in winter respiratory lesions are greater compared to other seasons, probably due to worst climate parameters and ventilation settings on the farm [56,62].

Another important factor that can affect the comparison between studies on welfare-related issues is the difference between countries in the application of animal welfare policies, with particular regard to the ban of the tail docking and provision of enrichment material as stated by Council Directive 2008/120/EC [6]. In fact, in many countries, the majority of the pigs are routinely tail docked, with just four countries (Norway, Sweden, Finland and Switzerland) that banned routine tail docking [93,94]. This difference can be another obstacle when comparing results from data collected in different countries, regardless of the scoring methods used, in consideration of the fact pigs with undocked tails tend to have more frequently severe lesions compared to docked pigs [95,96]. In fact, the tail lesions prevalence, with reference to severe tail lesions, varies greatly among studies, ranging from 1 to 2.5% in studies on docked pigs [9,10,19,24,39] and from 6.3 to 9.3% in studies on undocked pigs [41,95].

Tail-lesions and skin lesions are generally considered as “iceberg” indicators of animal welfare reflecting on-farm situation; however, assessment of skin lesions at abattoir needs to be performed with caution. In fact, skin damage can occur on farms, especially during the finishing stage of production, but it can also occur during the transport and the lairage at abattoir [46]. During these steps, pigs may express fighting behavior during mixing of pens, loading and unloading, and introduction into the restrainers [97]. The extension to which skin lesions may be representative of on-farm welfare issues has been explored by Carrol et al. [18]. In this study, pigs housed in controlled conditions were followed through their productive life, and both tail and skin lesions were evaluated in each pig on the farm and at the abattoir. The authors concluded that individually severe skin damage occurring up to 11 weeks prior to slaughter remains visible on pig carcasses, although such lesions may appear mild on the carcass, despite they were classified as “severe” during the rearing phase. On the other hand, tail lesions appeared to be visible on the carcass regardless of the time when the lesions occurred during the lifetime of the pigs. Since skin lesions can induce bias in the determination of welfare status at the abattoir, more studies are required to determine the proper scoring method to assess them.

Apart from the above-described weaknesses of abattoir-based measures, it is to be noted that farmers can use different strategies to reduce the number of pigs with welfare issues that are sent to the slaughterhouse; for instance, pigs with severe welfare conditions can be sent to a different establishment or, in extreme situations, they can die or be euthanized on farms [10,39]. Therefore, pigs with such conditions would not be seen at the slaughterhouse, and this could result in an underrepresentation of the real situation of pig welfare status [98]. Finally, it is important to note that the assessment of gastric lesions at the slaughterhouse may be difficult to achieve from a practical point of view [99]. In fact, the examination of gastric mucosa must be performed separately from the slaughter line, requiring the opening of the gastric wall along the large curvature and the removal of the content prior to its evaluation [77]. These actions normally need an extra observer, which can come at an extra cost. Therefore, the assessment of gastric lesions, despite associated with welfare issues, cannot be performed routinely.

## 6. Future Perspectives

Currently, the different scoring methods utilized to assess swine welfare at the slaughterhouse level makes the comparison between studies sometimes difficult [26,92]. In addition to this, a certain rate of discrepancy in the assessment of lesions between observers may not provide reliable information, as previously reported [60]. There is a growing interest in overcoming such obstacles by using automated inspection systems [50]. These methods use convolutional neural networks (CNNs) to designate pathological conditions probabilities to image locations. Such images of probabilities can be converted in a heat map, which is submitted to a further classification, based on statistical methods, that decide whether to assign a certain pathology to an organ or not [50]. CNNs-based systems can be trained in order to detect and analyze a specific part of the animal body [100]. Some CNNs methods have been already developed to score pericarditis and ascaridiosis [50], pleurisy [101] and tail lesions [100].

Additionally, a machine-learning methodology was also implemented by Sanchez-Vazquez et al. [92] to investigate the relationship between different conditions (EP-like lesions, pleurisy, milk spots, hepatic scarring, pericarditis, abscess, pyemia, tail damage, papular dermatitis) collected at the slaughterhouse through the BPEX and WPS schemes.

The development of camera-based systems could allow a better standardization and validation of the results obtained from pig welfare assessment, as advocated by Valros et al. [26]. As far as antemortem activities are considered, the monitoring of the skin temperature of pigs at the slaughterhouse was also suggested as a method to detect pigs with severe tail lesions, but also to find the early onset of severe diseases such as the African Swine Fever [102,103].

## 7. Conclusions

Assessment of welfare in pigs is an important issue that requires reliable and valid indicators [24]. The monitoring of welfare outcomes at the slaughterhouse permits to obtain crucial information that allows farmers and veterinarians to eventually implement specific interventions and/or to adjust on-farm practices based on the welfare outputs [51]. Moreover, this information could be useful for the competent authorities when deciding to enforce animal welfare legislation. In addition to this, such a welfare scheme could be applied systematically and continuously at a less cost than on-farm welfare assessment: however, longitudinal studies are required to evaluate the capacity of the recording of carcass lesions over a long period of time [39].

The methods used to score the different lesions in pigs at the abattoir are different between studies, whether they involve the scoring of body lesions [10,21] or other organs [60], thus making the comparison between studies more complicated.

In order to increase repeatability and reproducibility of the data collected at the slaughterhouse, an attempt to benchmark a scheme comprehending abattoir-based measures should be implemented at the European level. The above-mentioned scheme should take into consideration several factors, such as the different workload and speed lines between slaughterhouses, as well the validity, repeatability, reliability and feasibility of the welfare indicators included [104].

## Figures and Tables

**Table 1:** Scoring methods for body lesions.

Lesion Type	Scores Description	References
Tail lesions		
	0—No evidence of tail biting 1—Healed or mild lesions 2—Evidence of chewing or puncture wounds, but no evidence of swelling 3—Evidence of chewing or puncture wounds with swelling and signs of possible infection 4—Partial (or total) loss of the tail 5—Total loss of the tail	[10,39,40]
	0—No visible lesions 1—Skin perforated with reddish discoloration 2—Skin perforated with reddish discoloration and loss of skin (dented skin) 3—Skin perforated with brownish or blackish discoloration and loss of skin (dented skin) CL—Complete loss of the tail up to the tail base with perforated or healed skin surface	[30]
	0—No injury 1—Swollen 2—Small sore or wound 3—Small sore or wound and swollen 4—Major sore or wound 5—Major sore or wound and swollen	[41]
Skin lesions		
	0—No visible skin damage, only one lesion greater than 2 cm or lesions smaller than 2 cm 1—Between two and 10 lesions greater 2 cm 2—Any wound that penetrates the muscle tissue, or more than 10 lesions greater than 2 cm	[12]
	0—Up to one lesion 1—From one to five lesions 2—More than five lesions or any wounds which penetrate the muscle	[23]
	0—None or little superficial damage 1—Some superficial damage clearly signed up to three short (2–3 cm) and deep lesions 2—Clear deep and long damage (>3 cm), including much superficial damage or circular areas 3—Much deep damage	[42]
	0—No lesions 1—One small (approximately 2 cm) superficial lesion (not penetrating the skin) 2—More than one small, superficial lesion or just one red (deeper than score 1), but still superficial lesion 3—One or several big (2–5 cm) and deep (a lesion penetrating the skin) lesions; if deep; only one single lesion; if not so deep; several red lesions 4—One very big (>5 cm), deep and red lesion or many deep, red lesions 5—Many very big, deep and red lesions covering the skin area (Red appearance = fresh lesion; no red appearance = healed lesion)	[18]
Bursitis		
	0—No evidence of bursa/swelling 1—One or more small bursae (comparable in size to a grape, 1–2 cm of dimension) 2—More than one small bursa on the same leg, one very large bursa (comparable in size to a tangerine, ≥7 cm of dimension) or any eroded bursae	[14]

**Table 2:** Scoring methods for pluck and gastrointestinal systems lesions.

Type of Lesions	Scores Description	Maximum Score	References
Lung lesions			
	Each lobe is assigned with a score from 0 to 4 according to the following classification: 0—No lesions 1—Lesion affecting < 25% of the lobe surface 2—Lesion affecting 25–49% of the lobe surface 3—lesion affecting 50–74% of the surface 4—lesion affecting ≥ 75% of the surface	28	[61]
	The percentage of each damaged lobe is multiplied by its relative size. The cranial lobes and the accessory lobe have a relative size of 0.10, while the caudal lobes have a relative size of 0.25. This scoring method is based on the “rule of ten” (5*10 (sum of caudal lobes maximum scores) + 10+10+10+10+10 (maximum score for the other lobes))	100%	[60,68]
	The percentage of each damaged lobe is multiplied by its relative weight and then summed to provide the total weight percentage of the altered lung. Relative weight of the lobes: right apical lobe = 11%, right cardiac lobe = 10%, right diaphragmatic lobe = 34%, left apical lobe = 5%, left cardiac lobe = 6%, left diaphragmatic lobe = 29%, intermediate lobe = 5%	100%	[65]
	The scoring of the affected lobes is applied by means of 0–10 points or 0–5 points, depending on the lobes. The cranial and medial lobes have a maximum score of 10, while the caudal lobes have a maximum score of 5.	55	[69]
Pleural lesions			
	0—No pleural lesions 1—Cranioventral lesions, pleural adherence between lobes or at their ventral border 2—Dorsocaudal unilateral focal lesion 3—Bilateral lesion of type 2 or extended unilateral lesion 4—Severely extended bilateral lesion	4	[72]
	APP index: ((frequency of pleuritis score ≥ 2)*(mean pleuritis score of the animal with score ≥ 2)	Non-applicable	[72]
	1 point for pleurisy affecting the cranial area of the parietal pleura; 2 points for pleurisy affecting the middle area of the parietal pleura; 3 points for pleurisy affecting the remaining caudal area of the parietal pleura	12	[73]
Gastric lesions			
	0—No lesions 1—Hyperkeratosis, 2—Erosion and/or mild ulcer 3—Severe ulcer	3	[82]
	0—Shiny white squamous epithelium, 1—Parakeratosis of pars oesophagea and thickened epithelium, 2—Erosion of squamous/glandular junction and start of ulcers, 3—Developed ulcers, hemorrhage and stenosis present	3	[83]
	0—Normal mucosa, 1—Mild hyperkeratosis covering less than 50% of the surface 2—Severe hyperkeratosis covering more than 50% of the surface 3—Hyperkeratosis with few erosions 4—Hyperkeratosis with several erosions 5—Hyperkeratosis with many erosions or ulceration	5	[84]
	0—No lesions 0.5—Slight signs of hyperkeratosis 1—>50% of pars oesophagea covered, 2—>75% covered 2.5—>75% covered and erosion visible, 3—Gastric ulceration in the pars oesophagea and/or stricture at the cardia	3	[85]

## Data Availability

No new data were created or analyzed in this study. Data sharing is not applicable to this article.
